# Thinking with ‘lexical’ features to reconceptualize the ‘grammar’ of schooling: Shifting the focus from school to society

**DOI:** 10.1007/s10833-020-09400-4

**Published:** 2020-08-13

**Authors:** Steven J. Courtney, Bryan Mann

**Affiliations:** 1 University of Manchester, Oxford Road, Manchester, M13 9PL UK; 2 University of Kansas, Lawrence, KS 66045 USA

**Keywords:** Educational change, Grammar of schooling, Lexical features, COVID-19

## Abstract

Achieving changes to education practices and structures is a significant issue facing reformers internationally, and researchers have confronted how such changes, and the conditions for these, might be conceptualized. These issues resonate particularly as researchers grapple with imagining a post-COVID-19 landscape where social and educational norms may change. Tyack and Tobin, in their 1994 article ‘The “Grammar” of Schooling: Why has it been so hard to change?’ argued that several features of the American education system are so persistent as to warrant being understood as the ‘grammar’ of schooling. In this article, we reconceptualize this ‘grammar’ by taking seriously Tyack and Tobin’s insistence that ‘grammar’ organises meaning. Starting here, we argue that what they took to be grammatical features are the products and not the producers of meaning. We draw on the cases of the United States and England to argue that four international discourses have performed this meaning-making work: industrialization; welfarism; neoliberalism and neoconservatism. These are the ‘grammars’ of schooling—and of society. Their discursive products, including age grading and sorting into subjects are, we suggest, ‘lexical’ features that express the grammar. We use lexical features to explain the multi-directional interplay between discourse and educational feature: the lexical may endure longer than the grammatical, changes to which may be effected and/or legitimated through appealing to a lexical feature. We conclude by outlining key implications for realizing and conceptualizing educational change, including for a post-COVID-19 landscape.

## Introduction

The COVID-19 pandemic has ended lives and disrupted ways of living. In this context, urgent questions are being asked in fields including, but not limited to education about which current practices, beliefs and structures might be kept, which discarded, and which re-thought. For instance, Courtney et al. (2020) highlight five contemporary education-related claims that the pandemic has revealed to be myths, including the sufficiency of teacher/leader efficacy in overcoming all effects on pupil achievement of disadvantage and the primacy of the individual in education provision and policy. Azorín (2020) puts it bluntly: ‘the post-pandemic schooling universe has two possibilities open to it: a return to traditional education as usual or a transformation towards another education’ (p. 1).

The education field is clearly at a moment of potential paradigm shift, yet little recent work has been undertaken to explain how such shifts are best conceptualized, to problematize the extent to which current conceptualizations are fit for purpose or to propose alternative thinking tools that might better enable understanding of significant educational change. COVID-19 is creating a space to remake education, and so the stakes are uncommonly high. We intend—and the field needs—to interrogate notions of what persists and what may evolve, be reformed or ended in education, and, importantly, why this might be so.

Tyack and Tobin originated the claim in their seminal 1994 article in the *American Educational Research Journal* that education systems have persistent features, which they characterized as constituting a ‘grammar’. In the article, the authors define the grammar of schooling as “the regular structures and rules that organize the work of instruction” (p. 454). Tyack and Tobin describe the American grammar of schooling as consisting in, for example, “standardized organizational practices in dividing time and space, classifying students and allocating them to classrooms, and splintering knowledge into ‘subjects’” (p. 454).

Tyack and Tobin’s argument has greatly influenced the field of education research and scholarly thought: a Google Scholar search of the article reveals 1605 citations as of July 2020. The continuing appeal of a schooling grammar also is evidenced through conference panels with the theme and dedicated special issues. For example, Mehta and Datnow’s (2019) special issue call is typical in framing the issue around the need to ‘move away’ from an industrialized model of education that subsequent waves of reform have failed to dislodge.

That such calls are necessary speaks to how challenging the task is of understanding why reforms have failed to change the perceived grammar of schooling. Tyack and Tobin (1994) provide few clues in their article, whose contribution is predominantly descriptive in the way in which it identifies and elucidates the problem, but stops short of explaining why grammars might exist or persist. Both the fundamental conceptualization of the grammar of schooling and the lacuna concerning how it might be explained are largely accepted in the literature, which focuses on proposing educational approaches that are potentially disruptive to an unproblematized and unexplained grammar (e.g. Martínez Arbelaiz and Correa Gorospe 2009); or which position this grammar as an intractable obstacle to reform (Lefstein 2009).

We suggest that Tyack and Tobin’s grammatical metaphor has acted as an anti-heuristic for the field: it closes down debates and thinking through its implacable, fatalistic emphasis on *what is*, rather than on *why* this is. This tends to reduce scholars’ engagement with the grammar of schooling to issues of ontology, where any sign of shared features with the extant simply confirms the grammar (see e.g. Marsh et al. 2020). Or, conversely, fairly localized practices and/or cultures that contradict the accepted grammar are constructed as a ‘new grammar’ with all the systemic significance of that metaphor, but with an unsatisfactorily explained mechanism for achieving it (e.g. Mehta and Fine 2019). And of course, even the task of description, which underpins the grammatical metaphor, implies a degree of interpretation, and Tyack and Tobin’s contribution does not particularly help scholars in this interpretive work. In Bucharová’s (2019) research in the post-authoritarian contexts of the Czech Republic, Poland and Portugal, for example, identified practices are ontologically elevated to the status of the grammatical simply through deploying the metaphor, limiting analytical and explanatory power.

In this article, we therefore intend focusing not only on substantive changes, but on the meaning of those changes. Exemplifying the former, both in the United States and internationally, is the rise of so-called autonomous, state-funded schools. Structurally homologous versions of these types of schools exist globally. For example, there are charter schools in the United States, concession schools in Colombia, academies in England, and friskolor in Sweden, among others. These types of schools are part of a wider trend to market-based ‘solutions’ to solve what are claimed, erroneously (Ravitch 2013), to be the intractable problems of public education. Another contemporary element of these solutions consists in the reification of a leadership ‘caste’ or class. Such leaders are deemed to possess what are discursively constructed as the unique skills and knowledge to exploit the autonomy required by the market (Gunter 2012), despite this autonomy being largely illusory (Salokangas and Ainscow 2018).

The COVID-19 pandemic, along with this emergence of new schooling models and shift toward educational privatization, means that now is the ideal moment to ask whether the notion of the ‘grammar of schooling’ still applies. Does this term capture past events and current trends? If not, how can we redefine it so it is better able to capture 21st education reform or cataclysmic, extrinsically derived change?

We aim in this article to provide an explanation for the persistent features of schooling, and thereby to complement and build on Tyack and Tobin’s (1994) original conceptualization. Through our analysis, we undertake a reconceptualization of the ‘grammar’ of schooling in order to shift the concept away from persistent features and towards the mechanisms responsible for those features implied through the linguistic metaphor of grammar. We argue that grammar brings meaning to persistent features, and so is external to those features. To exemplify schooling grammars, we identify discursive mechanisms rather than the reified product of those mechanisms, which, we suggest, is where the field has focused up to the present.

Our repositioning of grammatical features creates a new conceptual space for our new concept of *lexical* features: these are often durable, and sometimes more durable than grammatical characteristics, but they *express* the meanings suggested through the grammar and *do not create* them. We argue that thinking with our new concept of lexical features, and how it interplays with the grammatical, enables new ways of understanding why some features of school systems might persist, even where their underpinning logic has shifted. Our use of lexical features also reflects and encourages precision in naming what exactly has changed, or not, and hence re-positions the scholarly conversation on the appropriate object of analysis. Our expanded conceptualization of the ‘grammar of schooling’ exceeds the education system of any one nation, and so we draw on the cases of the United States and England in order to illuminate the deeper, shared structures of two nations with many common features and dispositions to education-policy development (Whitty 2016), and which export education policy internationally through processes of borrowing and travel. The rich conceptualizations we provide here will enable international engagement, either through recognition or refutation.

Our contribution to the field is conceptual through a new metaphorical understanding of grammatical and lexical features of schooling; it is explanatory through how we deploy these terms to analyze the reasons why schooling features persist or change; it is heuristic through its re-framing of processes of educational change; and it is predictive through articulating the (grammatical) conditions under which (lexical) changes must happen in order to persist. The analysis and arguments move the field to new and productive conceptual terrain concerning why certain features of schooling persist and how they might be understood.

### Reconceptualizing the ‘grammar of schooling’

Tyack and Tobin (1994) assert that ‘practices like age-graded classrooms structure schools in a manner analogous to the way grammar organizes meaning in language’ (p. 454). Reflecting upon this statement leads us to argue that age-graded classrooms and the division of knowledge into subjects, *inter alia*, represent not the producers, but rather the reified products of putatively grammatical processes. If, as Tyack and Tobin (1994) argue, the object of analysis is that which ‘organizes meaning’ (p. 454), then we suggest that in order to identify the grammar of schooling, we need to refocus the analysis at the level of discourse.

The grammar of an education system, insofar as it is meaning-making, therefore relates not to persistent structures, but to the *causes* of those ‘structures’, as in the discursive architectures that provide meaning in human relations, praxis and organizations. We are arguing for a shift away from Tyack and Tobin’s (1994) focus on the organizational and towards the discursive as the definitional differentiator. We suggest that Tyack and Tobin’s organizational focus produced an unnecessarily parochial analysis, in which their consideration of so-called grammatical features did not extend beyond one country, the United States. In contrast, an explanatory grammar which structures organizations is likely to have international features that require naming and taking into account.

In our analysis, organizational practices derive from and are attributed meaning through the overarching discursive grammar and are not reducible to it. Our argument reorients the scholarly conversation away from equating the grammar of schooling with structures and organization, which we insist reflect and do not comprise grammars. Structures such as batching can endure over long historical periods. However, this can conceal how underlying belief and therefore motivation systems have shifted, and so for us, in an important departure from Tyack and Tobin’s conceptualization, *grammatical features may actually be of shorter duration than the structures they engender*. Owing to features such as institutional inertia (DiMaggio and Powell 1983), we suggest that characteristics of a social institution change only if the dominant, exogenous grammar forces them to do so. Old expressions will persist unless contra-indicated by an emerging discourse and new expressions emerge to align with that discourse.

To make these arguments, we define a ‘grammar’ in schooling as a discourse or ideology that acts as the guiding structure across social institutions at a given period in a society because grammars occur at the discursive level, not the organizational. We have identified four grammars that influence schooling across the United States/England: industrialism, welfarism, neoliberalism and neoconservatism (see Table 1). These are widely addressed in the literature as discourses, however, we see them as constituting *grammars of schooling* when the following two conditions are fulfilled. First, where understanding of these four is both discursively shared and (more-or-less) internally coherent. Second, where they are considered in relation to the following domains: objectives of schooling, mechanisms and discursive legitimation. The mapping in Table 1 enables the identification of durable grammars and how these relate to educational movements.

**Table 1:** The four grammars of schooling in the United States and England

	Industrialism	Welfarism	Neoliberalism	Neoconservatism
Objectives of schooling	Efficiency	Equality	Economic growth	Elite reproduction
Mechanisms	Scientific Management	Purposive Mixing; Child-centered pedagogies	Marketization; Innovation	Hierarchized differentiation
Discursive legitimation	Efficiency	Fairness; Common good	Autonomy; Freedom; Choice	Eugenics

We labeled the grammars in Table 1 to elucidate how social discourse beyond schooling informs the expressions of organizational structure within schooling. Other scholars may contest the precision of these labels in describing social periods and, indeed, for our purposes they do reflect generalizations for large portions of 20th century history, and may apply differently or not at all in other countries. Our goal is not the precise labeling; rather to show how using these labels captures larger social discourse, which in turn shapes the organizational structures of schooling.

To make our argument, we have identified industrialism, welfarism, neoliberalism, and neoconservatism as distinctive discourses prevailing and sometimes recurring through various stages of 20th century policymaking and social life, although chronologically some of these overlap, particularly the latter two.

Industrialism came in the early part of the 20th century and centered public discourse on scientific management and the efficient allocation of resources, or Taylorism (Gray 1993). Workers were conceptualized and treated as cogs in a machine: indeed, the machine provided the metaphor for this discourse, whereby, for the sake of efficiency, humans’ agency and humanity were structured out of the production process. Efficiency consequently supplied both discursive legitimacy for and objective of the industrialist discourse. We argue that industrialism comprises a distinct grammar in creating social meaning in contexts beyond its industrial provenance, ranging from culture (see, for example, the factory scene in the 1936 movie “Modern Times”, starring Charlie Chaplin, and the 1927 silent movie, “Metropolis”, directed by Fritz Lang); through to how the Holocaust was predicated on industrial processes to effect genocidal murder.

Welfarism was predicated on Keynesian economics and, in its educational form, had equality as its objective. Education was understood as a common and public good: this was legitimated discursively through appeals to fairness and equal treatment, and operationalized through a focus on purposively mixing children within provision and on child-centred pedagogies. The structural focus was on non-selective, state-arranged education in both primary (elementary) and secondary (high school) phases—such policies might be driven from the center, from more localized jurisdictions, or even from grassroots practice (see e.g. Crook 2002). The evolution of this grammar, which manifested in education partly as progressivism, both produced and derived from distinctive local/national histories and forms, yet these localized forms spoke to a common western project ‘that sought the alleviation of pain and suffering and the promotion of moral and intellectual advancement’ (Reese 2001, p. 3): in realising it, ideas were mutually exchanged and reinforced across borders. Or, as Reese (2001, p. 5) puts it: ‘American Progressivism was literally the child of Europe’, and certainly, thinkers such as Johann Pestalozzi were foundational. Yet welfarism was never so dominant a grammar in the United States or in England as in other countries, particularly in Europe (see Arts and Gelissen 2002), or indeed so dominant as the grammars that preceded and succeeded it. This has made lexical items predicated upon it more amenable to change, either back to an earlier form or to a new form.

Originating intellectually in ideas by, *inter alia*, Hayek (1944) and Friedman (2009), the objective of neoliberalism is national economic growth, and as Harvey (2005) notes, it constructs individual responsibility for making rational choices within a market as the most efficient mechanism for organizing not just resources, but improving ‘human well-being’ (p. 2). Neoliberalism is legitimated through imbricated discourses of autonomy, choice, and freedom. Harvey (2005) identifies the years 1978–1980 as ‘a revolutionary turning point in the world’s social and economic history’ (p. 1) owing to the adoption (in varying forms) of neoliberal tenets by multiple key political and economic actors internationally: Margaret Thatcher in the UK, Ronald Reagan and Paul Volcker in the United States, Deng Xiaoping in China, and Sergio de Castro in Chile. Unlike the classical liberal subject, the neoliberal subject is *not* left alone, but rather is the object of intense, yet often indirect steering mechanisms whose objective is the creation of *homo economicus*, a rational, self-actualizing and individually responsibilized actor who relates to others in a marketized suite of frameworks. We follow hundreds of scholars who have located contemporary education policy in a neoliberal context (e.g. Apple 2004, 2011; Blackmore 2006; Ball 2003; Gunter 2012; Ravitch 2013; Whitty 2016). However, we re-frame neoliberalism for the purposes of our argument as a grammar that organises meaning in educational arrangements, and so supplies the logic behind such statements as this, from former US Secretary of Education, Arne Duncan, ‘We have to educate our way to a better economy. Our children are as smart, talented, committed, entrepreneurial, and innovative as children anywhere in the world’ (2010, np).

Importantly, proponents of neoliberal policies still claim a social-justice motivation: however, this conceptualization of social justice is thin, since as Blackmore (2006) notes, ‘markets, through individual choice, were [intended] to distribute equity. This discourse of choice was rights rather than needs based, informed by neo-liberalism’s competitive individualism rather than collective interests of social liberalism’ (p. 190).

Neoconservatism recalls earlier forms with similar objectives, mechanisms and modes of legitimation. Today, it is not found discretely from neoliberalism in the United States or in England, but is in what Apple (2011) calls a ‘complicated alliance’ (p. 22) with it. This discourse seeks to realign society with traditional hierarchical structures in order to reproduce elite interests, and uses neoliberal mechanisms to achieve and maintain these hierarchies. For instance, white, middle-class parents may choose schools whose pupils are socio-economically and/or racially similar to their child (Ball 2003), and so a key structural outcome of neoconservative education policy is hierarchized differentiation (see Courtney 2015a). Increasingly, however, arguments for according better, discrete provision for the wealthy are being legitimated using a discourse of eugenics, following the argument that the genomically well-endowed are more likely to succeed in life, and so wealth is both hereditary and inherited, and correlates with whiteness (for arguments for and against using genetics to inform public policy, see Plomin 2018; Saini 2019 respectively).

Using these descriptors of social discourse, we provide an analysis that repositions what has been understood previously as grammatical features. We understand them as products rather than producers of the structuring elements we have outlined. Consequently, we need a new categorical dimension to explain these products, and so here we present and discuss what we are calling lexical features of schooling.

### The lexicon of schooling

A lexicon is vocabulary used in accordance with the rules of grammar, which the lexicon expresses and reveals. In language, a grammar helps constitute and organize meaning, while a lexicon provides the means to understand the organizing principles constituting the grammar, but are not themselves those organizing principles. Identifying a single word does not necessarily help one understand a grammar; rather a set of words allows for understanding because it enables perception of the underlying grammatical structure. Lexicons are characterized by both stability and change. Some words persist, perhaps in mutated forms, and at times appear in multiple languages, while other words emerge and fade in short periods of time (Deutscher 2005). Considering only words that persist gets one no closer to understanding the underlying logic or rules, this persistence notwithstanding.

Describing certain features of schooling as lexical rather than grammatical is useful in understanding school reform because it illuminates why certain features of schooling persist. This framing also characterizes what has potential to change, and allows us to posit and explore the following novel interpretation: that while a certain pattern of practice may remain consistent at two time points, the meaning of these practices changes (examples will be discussed later in this article). Since lexical expressions reflect rather than produce the underlying grammar, lexical changes are more easily observable than grammatical changes, persisting for as long as they are compatible with the contemporaneous, structuring grammar.

The causal relationship is not unidirectional, however. We suggest that underlying grammatical features produce lexical features that, in turn, draw on and are legitimated by grammatical features. Indeed: some grammatical changes are made possible precisely by appealing to a persistent lexical item. So, for example, the shift from a welfarist to a neoliberal/conservative grammar did not preclude necessarily any shift away from teaching discrete subjects. The lack of any observable change in the schooling structure of subject teaching concealed the extent to which the significance of the underlying rationale had changed. In fact, the change in grammar to neoconservatism actually strengthened subject teaching: elite reproduction happens largely through transmitting arbitrarily privileged knowledge through the curriculum (Apple 2004; Bourdieu 1990). Subject teaching had only survived welfarism because it was not contra-indicated by that grammar, and not because it was a particularly good fit for it. In our new framing, grammatical does not simply mean most persistent, it means the element that ‘organizes meaning’.

Following this argument, perhaps owing to institutional inertia or other institutional forces (DiMaggio and Powell 1983), not only will lexical expressions thrive in situations where they best reflect the grammar, but also they persist in situations where they do not explicitly violate the logic of a new grammar, even if they are not best representing it. This explains why some lexical features remain while grammar shifts. Indeed, many lexical features of schooling remain durable thanks precisely to their ability to connect with multiple grammars.

Tyack and Tobin (1994) argue that certain features of schooling, such as age-graded schooling, batched classrooms, lecture style teaching, and tracking are grammatical. We contend that these features are lexical. While these features have remained consistent during the 20th century, viewing these consistencies through a lexical lens allows us to broaden the scope in our explanations of why these elements endure. The features Tyack and Tobin discussed did not change because of their inherent compatibility with multiple grammars, though they themselves were not grammars.

A key element that the ‘grammar’ argument also failed to capture was those features of schooling that *have* changed during the 20th century. These include expanding enrollments, changes to enrollment organization, the increased standardization of curricula centered on teaching to high-stakes testing, and the differentiation of governance, among others. We argue that those features that did change help us identify and explain the shift occurring to the current grammar of schooling and consider where this shift has led and will lead education systems in the United States, England and beyond.

### Reconceptualizing educational change using ‘lexicon’ and ‘grammar’

To exemplify and elucidate our argument, we explore below four lexical expressions, two of which Tyack and Tobin discuss, and explain how they relate to the four grammars of schooling we identified during the 20th and 21st centuries: industrialism, welfarism, neoliberalism, and neoconservatism.

Our first two cases, age-graded batch processing of students and sorting schooling into subjects, were selected because they were raised by Tyack and Tobin. We agree that these features of schooling have not changed in either the American or English contexts, but we supplement the original analysis by theorizing why these features endure despite grammatical shifts.

We then examine the cases of school enrollment and testing, two features of schooling not explored by Tyack and Tobin. The former is a feature of both U.S. and English national systems that has shifted in many areas in practice and in all areas in meaning. The latter was a practice in education that has come to signify and underpin a new architecture of accountability, and so exemplifies a substantive grammatical shift overlain with a lexical feature that is superficially persistent, yet substantively re-purposed.

### Stable lexical expressions: Age grading

The first persistent lexical expression involves how schools process and move their students through the system. Tyack and Tobin (1994) labeled this process as age grading, meaning students are expected to move together in aged groups from grade to grade *en route* to the completion of their educational trajectories. This expression has remained constant throughout different periods and different grammars of schooling.

Age grading was produced from the grammar of industrialization. Moving students efficiently through the system is homologous to moving widgets through a factory. This style of management reflects the industrial mindset of assembling products by their core parts and shifting them through a system when they are ready to receive the next part. This feature, despite sporadic opposition, remains systemically endemic (Tyack and Tobin 1994).

A welfarist grammar is insufficiently disruptive to eliminate age grading. In this grammar, increasing a child’s grade level based on his or her age can act as the developmentally appropriate choice to make. While efficiency may not be the core feature of a welfarist grammar, attempting to include all students equally and equitably in a system does not violate the use of age grading. Schools could simply attempt to make larger, more inclusive age-based cohorts.

While a grammar that privileges a more egalitarian educational system could have produced different features than age grading, such as personalized learning based on ability to meet the needs of individual students, age grading nonetheless still aligns with the emerging welfarism during this period. For example, during racial desegregation movements in the United States, race was rightly understood as constituting a more important dimension of difference than age in terms of its susceptibility to being used to disadvantage groups of people. The welfarist grammar did not address the effects of ageism, particularly amongst the young, but rather sexism and racism. Changes to enrollments occurred based on these ideals, but age grading remained as a feature because the welfarist grammar did not test its legitimacy. In the United States, racial segregation was challenged and, indeed, until the end of the welfarist period the schools once most segregated by race achieved their highest levels of racial diversity—a trend that would change as the welfarist period ended (Orfield and Eaton 1996).

Age grading also endures within neoliberal and neoconservative grammars because, again, it does not violate their principles. Thinking about neoliberalism first, this grammar incorporates age grading within the marketplace. That is, educational consumers have been primed to understand what constitutes a legitimate and hence worthy school and so they are not obliged to change these preferences in the new grammar. Models without some of the core features of schooling from other eras, so long as they do not violate the current grammar, are likely to persist in the neoliberal era because consumers shop for what they know. It may be valued in the market precisely because it is traditional.

Likewise, age grading may support neoconservatism because it remains a suitable tool to control *certain pupils*’ learning trajectory. A key element to neoconservatism is finding mechanisms to reproduce existing social structures, but to do so as covertly as possible. Age grading reflects age-based social structures, but does not reflect class-based structures, and so provides an ostensibly neutral structure through which subtler means of elite reproduction may function (e.g. colonization of entire schools in wealthier communities and the use of private tutoring).

### A relatively stable lexicon: Sorting into subjects

Tyack and Tobin (1994) describe the provenance of this schooling feature as ‘a gift of 10 million dollars by Andrew Carnegie to provide pensions to retired college professors’ (p. 460). Whilst true of the US context, this does not explain why this feature became popular at around the same time in international locations unaffected by Carnegie’s grant, including England. We suggest that the answer lies instead in the feature’s fundamental provenance in the industrialization grammar that was coming to dominate throughout the west, and reflects the requirement of multiple nation states for their future citizens to learn knowledge deemed to be contemporaneously important (see Apple 2004, for a discussion of how contested this notion is) for their economic role in the industrial age and, for some, for their participation in democracy.

Tyack and Tobin’s assertion that this knowledge enduringly is taught in subjects, and this therefore constitutes a grammatical feature of schooling, represents a simplistic reading of the history of education. They note similarities in how things *were* in comparison to how things *are*, and characterize intermediary, differing arrangements as aberrations. We acknowledge the *stickiness* of these features, but argue instead that these intermediary lexical changes as well as our contemporary iteration do indeed reflect changes in the underlying grammar, but only *to the extent that the grammar itself became embedded*, and that there is no inherent reason why future grammatical changes will not shift this lexical feature again into one that is less adherent to subject-sorting.

By way of exemplification, we turn first to the shift from the industrial to the welfarist grammar in the *progressivist turn*, which as we have noted, was not so deeply fixed in American or English education systems as in others. However, there were concomitant changes to lexical features in these countries, including an increase in kindergarten provision (Reese 2001) and, in England particularly, a focus on delivering elementary, or primary education sometimes through integrated topics rather than subjects. Gillard (2018) notes that in the 1950s and 60 s, pioneering Directors of Education (Supervisors) in a few Local Education Authorities (LEAs, or Districts) promoted integrating the curriculum on planned occasions: this locates the dismantling of subject-based delivery as part of a progressivist project. During this period, LEAs enjoyed a dominant role over the central government in organizing and managing educational arrangements, which enabled greater experimentation but potentially limited the spread of novel lexical features.

As a further example, we draw on the nuanced case of the studio school. This is a sub-type of academy (charter school) that was created in 2010 for England by the UK Conservative-led Coalition Government (education being devolved to the four nations of the UK). Studio schools were part of a policy agenda not only to expand schooling provision in England, but also to differentiate and covertly hierarchize it: this agenda may consequently be seen to illuminate and reproduce the dominant neoliberal and neoconservative grammars (Courtney 2015a; McGinity 2015). We are using them as an example here because they offer an integrated, industry-approved and co-delivered curriculum as an integral element of their signature pedagogy. Studio schools were and are positioned in a crowded market of provision through this integrated curriculum in order to attract “certain”, i.e. lower-attaining students: this was further attempted by proposing additionally a limited curricular offer and lower exit qualifications than is usually the case in secondary schools. Now-defunct website advertising appealed explicitly to ‘students who are better suited to a more “hands-on” approach to learning’ (Studio Schools Trust 2011, in Courtney 2015b, p. 123), whilst simultaneously claiming that studio schools are non-selective. In effect, the selection was intended to happen through the branding, with potentially lower-attaining students recognising themselves in the advertising and self-selecting into inferior—or, as was claimed, more suitable—provision.

This did not happen. Twenty-six studio schools have closed or will soon close, with only 29 remaining open (Allen-Kinross 2018). The reduction in subject-based teaching in favor of an integrated, vocational-skills-focused curriculum was intended simultaneously to appeal to the needs of industry and to stand out in the market of provision. We argue that its failure was not because subject-based teaching is an enduring grammatical feature, but rather partly^1^ because this attempt at curricular integration came as part of a package that attempted to do the impossible, that is, to provide an apology for, even champion, education provision that is designed to be inferior. In other words, this lexical feature’s success is contra-indicated by the neoconservative grammatical imperative to function covertly and with the consent of the dominated. England is accustomed to educational segregation, with 163 grammar schools still operational, down from 1298 in 1964 (Statistics of Education 1964 HMSO 1965, p. 12, quoted in Gillard 2018). Champions of grammar schools often draw on their personal experience of advancing socially through attending a grammar school (see e.g. the former UK Prime Minister, Theresa May 2016) instead of the empirical evidence that shows how over a population, grammar schools do not help social mobility (Gorard and Siddiqui 2018). What is never championed is the destination of those who fail the grammar-school entrance exam^2^: the secondary modern, which is invisible in policy discourse. We are suggesting that supporting studio schools is analogous to making pro-secondary-modern arguments, and contravenes the grammatical principles of neoconservatism in being too overt for the subordinated to buy into. Teaching in or out of subjects is not the deciding issue here, and so this example speaks to the importance of the wider context in framing individual issues or features as key, or *grammatical*.

Finally in this section, we note that a great deal of ideological flexibility is permitted through the subject-based lexis, and so, following our tenet of persistence until contra-indicated, this lexical feature is perfectly able to express and reproduce the most recent neoconservative grammar with its focus on what are constructed as traditional, high-value subjects. For instance, Apple (2004) characterizes the attitude underpinning the shift to a neoconservative grammar in the following way:

If teachers and curricula were more tightly controlled, more closely linked to the needs of business and industry, more technically oriented, with more stress on traditional values and workplace norms and dispositions, then the problems of achievement, of unemployment, of international economic competitiveness, of the disintegration of the inner city, and so on would largely disappear, or so goes the accepted litany (p. xix).

We draw on Apple’s insights to note how sorting into subjects does not contravene this grammar: the requirement for schools to privilege technical knowledge may be operationalized primarily through privileging technical subjects and technical expertise.

### A Lexicon that has changed in meaning: Student enrollment

At the start of the 20th century, not all students were forced to enroll in schools and when they did, their placements reflected legal and social ideologies of racism and classism. Legal segregation served as a core feature to the US system in the industrial period. This was perceived as efficient for an industrial society at the time (though clearly and overtly racist) because overarching social structures meant placing students into the educational situation most likely to reflect their projected social standing, whereas those who did not enroll in schools went straight into the labor market (Patterson 2001).

During the course of the early part of the century and after industrialization, the legal dynamic and social ethos surrounding school enrollment changed, as state actions and shifting ideologies created systems aimed at hypothetically fostering more inclusive enrollment practices. These changes included the dismantling of racial segregation policy in the United States, and adding compulsory attendance laws. Not only was schooling available for children, it is a required part of childhood, and society began to expect that students would participate. Schools reached their highest point of racial integration and lowest gaps in achievement in the 1980s before neoliberalism emerged (Orfield and Eaton 1996).

The expectation of shared schooling failed to live up to its potential. As neoliberalism and neoconservatism expanded across the globe in almost immediate response to the welfarist ideology influencing educational systems in the middle of the century, so too have the patterns and mechanisms of school enrollment. Policy actors have infused market principles in many school plans, including charter schools in the United States and academies in England. These models expect school choice enrollment patterns to lead to competition and innovation (Hoxby 2001, 2003; Chubb and Moe 1990; Friedman 2009). Meanwhile, school enrollments remain differentiated by class and race (Reardon and Owens 2014).

Traditional enrollment models still align themselves with a current grammar of neoliberalism and thus are likely to persist with modifications. These modifications include the relationship between real-estate decisions and school enrollments. There is increasing reliance on school rankings in home purchases, reflecting a marketization of even traditional common school models; these practices increase segregation while altering the value of homes (Hasan and Kumar 2019; Lareau and Goyette 2014). Schooling has added lexical expressions that evidence a grammar of neoliberalism, while re-defining old practices to meet this grammar.

The evidence of re-defining comes in the amount of information and ranking available for traditional public schools. Students still become assigned in these models, but the current ideology expects parents to be informed shoppers of their school districts through choosing the available metrics of quality. In areas where low-income students do not have mobility through home purchasing, the neoliberal movement has provided in many instances schools of choice that low-income students can select.

This shift in enrollment aligns with shifts in grammar, especially since these movements align with growing neoconservatism. The ‘choices’ of neoliberalism align with the hierarchies of neoconservatism. The industrial period likely would not have offered this type of enrollment model because multiple options become less efficient. A continuation of the welfarist movement would have sought greater integration and sharing of resources. The current enrollment models in practice and meaning align with neoliberalism in that they allow for markets, and they align with neoconservatism because they allow for hierarchies: markets tend to be unequal in how students are placed.

### A lexicon that has changed in practice: Testing

Much of the grammar-of-schooling argument suggests that the core practices of teaching and learning, or the ‘technical core’ of educational practice has not changed during the 20th century (and now in the 21st century). While this is true in many regards, there is one core feature of classroom practice that has changed: student testing and accountability. This change has occurred in a way that tracks the changes in the underlying grammar.

The monitoring of student progress with multiple exams that occur at many points of the school year, taking much more time of instruction, is now a core feature of schooling in both the United States and England (Gorski and Zenkov 2014; West 2010). This schooling practice is now so entrenched that it would have to be considered as a ‘grammar’ in Tyack and Tobin’s original argument, though it makes little sense that this grammar would emerge in the system on its own. We suggest that the major movement to incorporate standardized testing into the educational practice in the classroom, in the technical core, has occurred because it is an emergent lexical expression of a shift in the grammar of schooling to neoliberalism and neoconservatism. While testing has existed for as long as schooling, the underlying meaning and significance of this testing has changed to reflect grammatical changes.

Testing was a feature of the industrial system in the United States: employed to sort children through IQ, it served as a mechanism of efficiency. This sorting was deterministic in constructing some individuals (and groupings, particularly racial) as inherently more intelligent than others, requiring the school system to efficiently organize classrooms to exploit perceived benefits of categorizing individuals according to measured intelligence (Ravitch 2001).

A key shift in the underlying grammar determining meaning in testing is captured and exemplified in the change from welfarist to neoliberal principles embodied in English schools as ‘Assessment for Learning’ (AfL). This concept was popularized thanks to a widely read literature review by Black and Wiliam (1998), “Inside the black box: Raising standards through classroom assessment”, which had a particularly strong section on recommendations for changes in practice. There is a tension in their elucidation of AfL: key tenets indicated an adherence to welfarist/progressivist principles. Kucey and Parsons (2017) make explicit the provenance of several AfL ideas in the work of John Dewey, albeit drawing on Chappuis (2009) rather than Black and Wiliam (1998). Examples shared by both exponents of AfL, and hence deriving from Dewey, include formative and ungraded feedback, pupil self-assessment, communication to discern understanding, and reflection.

However, these methods were intended by Black and Wiliam (1998) to be deployed in the service of a standards agenda that conflates education with examination success: this positions their contribution, if not the ideas themselves, firmly within the neoliberal agenda that was intensifying during the period in which they were writing. Testing as a technique to construct and measure easily quantified outputs of an education system has increased owing to its function as an expression of the neoliberalized grammar of schooling. It has become the dominant form of the broader, overarching descriptor, assessment, which was more amenable to multiple meanings. Testing also aligns with neoconservatism because tests allow a pseudo-meritocratic and market-legitimized mechanism to reproduce social elites. Testing has increased in use in educational practice to the point where it can be called a lexical expression of these movements, and it is pervasive enough to have reached the technical core of teaching and learning in the classroom. Importantly, and following the argument we have presented throughout, formative, child-centered assessment was inconsistent with the principles of the new grammars of neoliberalism and neoconservatism, and so was replaced.

### Reorienting the conversation

Our presentation and use of the new concept of lexical features, and our subsequent analysis, advances the scholarly conversation on the grammar of schooling by formalizing the role of some of the other factors in play that are unrecognized in Tyack and Tobin’s (1994) original theorization. We have argued that influence within these change processes is not unidirectional, but recursive, and so for instance, a lexical feature such as teaching by subject may simultaneously derive from an underlying grammar (industrialism) and legitimate new grammars (e.g. neoconservatism). In each case, this is because it fulfils the grammar’s fundamental instrumental objective, and yet equally, in each case this objective differs. Indeed, we suggest that this process of appropriative legitimation, where new sets of needs are met under the cover of the old, is a crucial way in which particular groups of people or particular institutions make the system work for them. It is easier to build upon extant structures and mold them to meet new requirements than to build new structures.

In our analysis, far from being ‘sticky’ or stubbornly persistent, we see features of schooling—lexical and grammatical—as being in constant flux and in continual interplay. Superficial endurance may mask a change in the underlying logic such that the significance of the social practice concerned may be changed out of all recognition. We argue that testing is one such example, where testing for admission to the next phase of learning; for establishing a child’s interests; or retention of a learning objective; for informing the next pedagogical decision; or to operationalize teacher/school leader accountability are fundamentally different practices serving fundamentally different objectives.

So what does this mean for enacting educational change? We identify several implications. First, new lexical changes are unlikely to succeed if they are contra-indicated by the underlying grammar. Attempts to enact such changes should therefore first concentrate on shifting the grammar. This is not impossible: we note the fundamental provenance of grammatical shifts often in macro-economic changes, another of which we are presently experiencing owing to COVID-19: these may affect societies internationally. However, these changes are not external to human agency, particularly at governmental-policy level, or through the media, and so we argue that major, or in our terms, grammatical changes need governmental support. This is not presently likely in an age of depoliticization, where the State has shifted the responsibility for the politics of policy-making onto quasi-statal actors and onto families in a sustained series of self-negating acts of privatization (see Gunter 2019). Changes that such actors may bring about in this paradigm, this *school*-*led system*, are inevitably lexical, and so are fundamentally concerned with reproducing the present grammars of neoliberalism and neoconservatism, using education as the mechanism. This is in itself a profoundly neoconservative act.

Related to this last point, our analysis implies that those heading up schools, constructed as *leaders*, who *exercise leadership*, are unlikely to effect grammatical change. They are somewhat better placed to bring about lexical change, but more likely through appropriative legitimation rather than through introducing an explicitly new lexical feature, since the atomization that accompanies individual schools’ ‘freedom” in a neoliberal grammar precludes the easy spread of positively disruptive changes across a system. This precisely contradicts dominant policy discourse in the United States and England (e.g. Duncan 2010; Gove 2011) and also contradicts how leadership is contemporarily constructed as concerning primarily change. Such constructions might be at school level, through a focus on, for example, transformational leadership (e.g. Leithwood and Jantzi 1990) or on delegation misrecognized as distributed leadership (see Gunter et al. 2013); or it might be at what is known as system level through ‘system leadership’ (Hopkins 2007), some of whose forms are better understood as a corporatized product of mergers and acquisitions (Courtney 2017).

We suggest therefore that appropriative legitimation is the most likely means of effecting change at levels other than at the statutory, where rather than attempting to change persistent features, new meanings might be attributed to them in order to achieve new purposes. This happens frequently at government level in developing and winning support for educational changes: we suggest that other policy actors too, including those in charge of schools and also teachers, can attempt this counter-discursively, where resistance to or mediation of harmful discourses may be required but challenging.

## Conclusion

Our analysis shows that reformers struggle to change what Tyack and Tobin term the ‘grammar of schooling’ because grammars run much deeper than schooling. Grammars are more deeply embedded in society and social institutions, while the practices in schooling reflect these deeply held ideologies as lexical expressions of grammar. Thus, true change to these practices requires a much larger shift to ideology.

This analysis means that in order to achieve substantive educational change, one of two strategies need to occur. The first is to see a change in a social ideology at a given point in time. The second is that otherwise, educational reform and substantive changes likely only emerge if they align with the contemporaneous grammar. For example, if one wishes to end class-based or race-based school segregation in a school movement driven by a neoliberal grammar, then one needs to either upend neoliberalism or find a strategy to use market-compatible solutions to achieve policy goals. The paradox with the latter strategy is that it likely ossifies the ideology, which in turn may make reform more difficult to achieve. No individual within an institution, nor any institution has ever changed the grammar of an education system without state support.

We opened this analysis with a focus on the continuing COVID-19 pandemic. Thinking about its implications using our thinking tool of lexical features in interplay with a redefined grammatical suggests the following. First, it is impossible to gauge accurately the extent to which a given event, however cataclysmic, signifies potential grammatical change, *whilst living through that event*. Second, whilst it presently feels as if everything has changed, in fact so far, only lexical change has occurred. Nothing about this pandemic changes the fact that many societies’ education systems currently function according to market principles, often with a large supplementary dose of conservatism. This is why most attempts to identify a shift in the grammar (but which are actually focusing on the lexical) do not succeed (see e.g. Marsh et al. 2020). Whilst it is possible to interpret the pandemic as a call for a common approach to education based on the public good, and as evidence of the concomitant failure of the individualistic market, it is equally important to recognize the widespread desire to *get back to normal*. So, as Azorín (2020) notes, much, or nothing may change: all depends on the durability of neoliberalism/neoconservatism.

Further, change may happen at the grammatical or lexical level. Concerning the former, we can start to imagine what new discourses or grammars might arise in a post-pandemic world. These could relate to public health over efficiency and the safety of all over the freedom of the individual. As such, based on our analysis, schooling arrangements may see new lexical traits reflecting this discourse. Of course, this would only happen if this discourse emerges as influential, which likely depends on the duration and impact of the coronavirus yet to be seen over the next several months.

Another future is also possible, in which only lexical change occurs. Private companies or institutions (in the UK, these might be constituted legally as charities and still make a profit) may exploit the crisis to reinforce, not replace the market. This is happening in England, where Hyman (2020), the Co-Director of a Multi-Academy Trust (comparable to a Charter Management Organization in the United States), has proposed superficially important changes to education for the post-pandemic landscape, but which his ‘social enterprise’ would be best placed to deliver. This illuminates how lexical changes may be offered up to protect deeper interests which are vested in the grammatical.

The implication of this key conclusion arising from our analysis is that effecting educational change is often profoundly undesirable to those involved. This is because lexical features serve to support, or at least not to undermine the overarching grammar, and this grammar organises social meaning. Grammars of schooling therefore underpin careers and identities (see Hughes et al. 2019), and constitute the field of power relations within which positions are taken, assumed and imposed (see Bourdieu 1990). From this perspective, the question of educational change is not so much technical as ethical and power-laden. What is needed is a new narrative to seize the moment for the public good and conjure the next grammar through prefiguration and imagination.

## Footnotes

1 Other reasons exist too, of course, including an out-of-sync transfer age and disproportionately poor standards.

2 Known as the 11+ examination.
